# Glycerol and Natural Deep Eutectic Solvents Extraction for Preparation of Luteolin-Rich *Jasione montana* Extracts with Cosmeceutical Activity

**DOI:** 10.3390/metabo13010032

**Published:** 2022-12-24

**Authors:** Aleksandra Maria Juszczak, Marijan Marijan, Lejsa Jakupović, Monika Tomczykowa, Michał Tomczyk, Marijana Zovko Končić

**Affiliations:** 1 Department of Pharmacognosy, Faculty of Pharmacy and Biochemistry, University of Zagreb, Marulićev trg 20/II, 10000 Zagreb, Croatia; 2 Department of Pharmacognosy, Faculty of Pharmacy with the Division of Laboratory Medicine, Medical University of Białystok, ul. Mickiewicza 2a, 15-230 Białystok, Poland; 3 Department of Organic Chemistry, Faculty of Pharmacy with the Division of Laboratory Medicine, Medical University of Białystok, ul. Mickiewicza 2a, 15-222 Białystok, Poland

**Keywords:** *Jasione montana*, Campanulaceae, antioxidant, collagenase, elastase, hyaluronidase, luteolin, natural deep eutectic solvents

## Abstract

*Jasione montana* is a plant from the family Campanulaceae rich in phenols with health-beneficial properties such as luteolin (LUT) derivatives. In this work, a glycerol-based ultrasound-assisted extraction method was developed and optimized for in total phenol (TP) and LUT content, as well as antiradical activity (RSA). The best conditions (glycerol content, temperature, plant material weight, and ultrasonication power) for the preparation of *J. montana* extracts richest in TP (OPT-TP), LUT (OPT-LUT), and having the best RSA (OPT-RSA) were determined. Furthermore, numerous natural deep eutectic solvents (NADES), containing proline, glycerol, betaine, urea, and glucose were prepared and used for the extraction of *J. montana.* Contents of TP, LUT, and RSA in the prepared extracts were established. Antioxidant and cosmeceutical activity of the prepared extracts was tested. The OPT-TP, OPT-LUT, and OPT-RSA, as well as the most efficient NADES-based extract, PG-50-TP, were excellent antioxidants and Fe^2+^ ion chelators. In addition, they were potent inhibitors of collagenase and hyaluronidase, as well as good significant anti-elastase and -lipoxygenase activity. The observed antioxidant- and enzyme-inhibiting activity of *J. montana* extracts prepared using environmentally friendly methods and non-toxic solvents makes them promising ingredients of cosmeceutical products.

## 1. Introduction

Products that are derived from natural sources, such as plants, are in special demand in the cosmetic market, due to the consumers’ perception of their increased safety and bioactivity. It is widely considered that they can prevent and delay skin aging and deterioration [1]. Indeed, an increasing number of studies shows that numerous plant extracts display high, almost pharmaceutical, efficacy, sensorial advantages, and safety. As such, they are ideal candidates for so-called cosmeceuticals, cosmetic products with performance characteristics that suggest pharmaceutical action. Even though the word “cosmeceutical” is a marketing rather than a legal term, it is often used in lay language because it reflects well consumers’ expectations [2,3].

To integrate botanical ingredients into cosmetic formulations, they must first be extracted from fresh or dried herbal material. Ideally, this is accomplished using non-toxic, biocompatible, and cost-effective “green” extraction techniques and solvents [4]. Glycerol (GL), a natural, odorless, nontoxic, and biocompatible liquid is one of the most widely utilized eco-friendly extraction solvents and important constituent in skincare products; it acts as a humectant and skin protectant [2]. GL is also commonly utilized as a component of natural deep eutectic solvents (NADES) that are defined as a combination of two or more natural compounds with hydrogen-bond-accepting and hydrogen-bond-donating properties. These mixtures possess unique physicochemical properties such as low toxicity, large solvency, low vapor pressure, and high biodegradability which define them as green and environmentally friendly solvents [5,6]. NADES are regularly used in extractions due to their ability to efficiently dissolve various types of natural bioactive compounds including flavonoids and other phenolic compounds [7]. Commonly used NADES components are GL, various sugars, such as glucose (Glu), and betaine (Bet), a quaternary ammonium salt of natural origin that may be effortlessly included in the final cosmetic products [8,9]. Proline (Pro) is also a commonly used NADES ingredient that provides solvents with beneficial physicochemical properties [10]. NADES are usually utilized as aqueous solutions. In addition to lower price, NADES-water solutions have better performance, and the solvent handling and application are simplified due to lower viscosity in comparison to the undiluted NADES [11,12]. GL and NADES containing non-toxic components may simply be integrated into the final product, making such extraction very advantageous from an energy-conserving point of view [13].

Numerous studies have shown that phenolic compounds, present in the plants and plant extracts, are desirable preservatives and functional ingredients in cosmetic products due to their antioxidant activity and their ability to impede numerous processes that negatively affect skin health and appearance [1]. For example, flavonoid luteolin shows excellent properties related to skin aging, wound healing, as well as inflammatory skin diseases, including psoriasis, contact dermatitis, and atopic dermatitis [14]. In addition, luteolin and its derivatives display promising anticancer properties against melanoma and other skin cancers [15]. Thus, luteolin and luteolin-rich plants might be promising candidates for the development of cosmeceutical preparations and products [14]. One of the plants especially rich in luteolin and its derivatives, such as luteolin 7-*O*-glucoside and luteolin 7-*O*-sambubioside, is Sheep’s bit (*Jasione montana* L., Campanulaceae) [16], a biennial or occasionally an annual garden plant, distributed in Europe, northern Africa, parts of Turkey, and in the north-eastern part of the United States. It is mostly found in warmer, sunny, or semiarid places in plains and mountainous areas [17]. As luteolin and its derivatives are among the major components of *J. montana*, it is not surprising that the plant shows an excellent cosmeceutical potential. For example, it was found that *J. montana* extracts may promote the viability and accelerate the migration of fibroblasts. Furthermore, luteolin-rich *J. montana* extracts were found to be able to modulate oxidant and inflammatory processes, as well as impede elastase activity [18]. Anti-melanoma activity of luteolin-rich extracts of *J. montana* was also confirmed [16].

Previous studies have demonstrated that luteolin and luteolin-rich *J. montana* extracts show numerous skin-related beneficial properties. Such promising cosmeceutical potential makes them excellent candidates for the development of cosmetic and dermatological products. Having in mind the importance of extraction conditions for the preparation of plant extracts rich in target compounds, the aim of this work was to optimize the extraction of *J. montana* and obtain extracts rich in luteolin and other phenolic compounds with high antioxidant and cosmeceutical properties. As biocompatibility is of utmost importance for the preparation of topical formulations, GL and NADES, as environmentally and skin-friendly solvents, were utilized for the extraction.

## 2. Materials and Methods

### 2.1. Plant Material, Chemicals, and Apparatus

*J. montana* was acquired in Puszcza Knyszyńska (Podlasie Province, Poland; 53°15′20.9” N; 23°25’41.5” E). The species has been authenticated based on the scientific literature and verified by Michał Tomczyk using referent literature [19]. A voucher specimen (No. JM-15029) has been deposited in the plant collection of the Department of Pharmacognosy, Medical University of Białystok (Poland). The enzymes and the HPLC standards (≥98.5%) were obtained from Sigma-Aldrich (St. Louis, MO, USA). Other reagents and chemicals were of analytical grade. Extraction was performed using Bandelin SONOREX^®^ Digital 10 P DK 156 BP ultrasonic bath (Berlin, Germany). A microplate reader (FLUOstar Omega, BMG Labtech, Ortenberg, Germany) was used for UV-VIS spectroscopic measurements. Agilent 1220 series HPLC instrument equipped with an autosampler, DAD detector, and a Zorbax Eclipse XDB-C18 (5 μm, 25 mm × 4.6 mm) column (Agilent Technologies, Santa Clara, CA, USA) was used for determination of the selected compounds.

### 2.2. General Extraction Procedure

Dried powdered plant material was suspended in 10 g of the appropriate solvent in a 50 mL Erlenmeyer flask, and extracts were prepared using ultrasound-assisted extraction (UAE). Following the extraction and subsequent filtration (Filtrak Qualitative Folded Filters Grade 6, 80 g/m^2^, Sartorius, Göttingen, Germany), the extracts were stored at −20 °C in the dark until use. The detailed extraction conditions for each phase of the extracts’ preparation are presented in Section 2.3. (Glycerol Extraction using 2-Level Factorial Design), 2.4. (Glycerol Extraction Using Box-Behnken Design), 2.5. (Extraction Optimization), and 2.6. (Extraction Using Natural Deep Eutectic Solvents).

### 2.3. Glycerol Extraction Using 2-Level Factorial Design

For 2-Level Factorial design (2LFD) six independent variables were selected for screening process as follows: glycerol content (X_1_, 10 to 90%, *w/w*), temperature (X_2_, 30 to 80 °C), extraction time (X_3_, 10 to 30 min), lactic acid content (X_4_, 0 to 5%, *w:w*), drug weight (X_5_, 0.2 to 0.4 g), and ultrasonication power (USP) (X_6_, 144 to 720 W). The extracts were prepared as described in Section 2.2. (General Extraction Procedure), while the detailed extraction conditions for each extract are presented in Table 1. Total phenol (TP) and luteolin content were selected as responses.

### 2.4. Glycerol Extraction Using Box-Behnken Design

After the detection of the statistically significant factors, Box-Behnken design (BBD) was performed using only the dependent variables that statistically significantly affected the dependent variables. Those were as follows: (−1 level, +1 level, unit) X_7_ = glycerol content (10, 90, % *w:w*), X_8_ = temperature (40 and 80 °C), X_9_ = plant material weight (0.3, 0.6, g), and X_10_ = ultrasonication power (432, 720, W). The extracts were prepared as described in Section 2.2. (General Extraction Procedure), while the detailed extraction conditions for each extract are presented in Table 2. TP, luteolin content, and radical scavenging activity (RSA) for 2,2-diphenyl-1-picrylhydrazyl (DPPH) radical were selected as the responses.

### 2.5. Extraction Optimization

In order to determine the optimal conditions for the preparations of the extracts with the desired characteristics, BBD results were analyzed and extraction optimization using response-surface methodology (RSM) and desirability function was performed. Three optimized extracts were prepared: OPT-TP (maximum amount of TP), OPT-LUT (maximum amount of luteolin), and OPT-RSA (lowest RSA IC_50_ value). The optimal extraction conditions were calculated, and the extracts were prepared as described in Section 2.2. (General Extraction Procedure), while the detailed extraction conditions for each extract are presented in Table 3.

### 2.6. Extraction Using Natural Deep Eutectic Solvents

NADES solvents were prepared using the modified heating method. The mixtures were kept at 75 °C for 24 h until a clear solution was obtained [9,20]. The following solvents were thus prepared (mixture components and their weight ratios are given in parentheses): 1BGG (Bet:GL:Glu, 4:20:1, *w:w:w*), 2BGG (Bet:GL:Glu, 4:5:1, *w:w:w*), GU (GL:Urea, 1:1, *w:w*), and PG (Pro:GL, 2:5, *w:w*). Each solvent mixture was prepared and diluted with water either in 1:2 (50%) or 1:4 (25%) ratio to obtain corresponding NADES-50 and NADES-25 solutions (e.g., PG-25 and PG-50). The extracts were prepared as described in Section 2.2. (General Extraction Procedure) for the extracts OPT-TP, OPT-LUT, and OPT-RSA. The detailed extraction conditions were identical to those described in Table 3, with the exception that NADES were used for the extraction instead of GL/water mixtures.

### 2.7. Spectrophotometric Determination of Total Phenolic Content

Total phenolic content (TP) was determined applying the modified Folin–Ciocalteu method, by mixing 80 μL of the diluted extract with 80 μL of Folin–Ciocalteu reagent and 80 μL of 10% sodium carbonate solution [21]. After 1 h the absorbance at 700 nm was measured and TP concentration was determined from the calibration curve and expressed as μg of gallic acid per mL of extract.

### 2.8. HPLC Analysis of Phenolic Constituents

All the extracts and the standards (0.2 mg/mL) were filtered through a 0.45 μm PTFE syringe filter (CHROMAFIL Xtra PTFE, Macherey-Nagel, Düren, Germany) before application. The solvents A and B were tertiary mixtures of water, methanol, and formic acid in the 93:5:2 (*v:v:v*) and 3:95:2 (*v:v:v*) ratio, respectively [22]. Solvents A and B were applied according to the following protocol: 0 min 20% B, 10 min 40% B, and 35 min 50% B. Separation was achieved at 40 °C and flow of 1.0 mL/min. The content of luteolin was determined at 270 nm. The peak assignment and identification were based on a comparison of retention times and UV spectrum of peak in sample chromatogram with that of the standard.

### 2.9. Radical Scavenging Activity

For determination of radical scavenging activity (RSA), methanolic DPPH solution (70 μL, 0.21 mg/mL) and 130 μL of the methanolic solution of the extract (sample) were mixed and incubated in the dark at room temperature for 30 min [22]. Methanol (130 μL) was applied as negative control and butylated hydroxyanisole (BHA) solution (130 μL) as the positive control. The absorbance was read at 517 nm and RSA was calculated using Equation (1):

(1) $$\mathrm{RSA}\;\left(\%\right)=\frac{A_{0}-A_{s}}{A_{0}}\times 100$$

where A_0_ is the absorbance of the negative control and As is the absorbance of the sample. RSA IC_50_, concentration of the extract that scavenges 50% of DPPH free radicals present in the solution, was expressed as μL of extract/mL of solution (μL extract/mL).

### 2.10. Fe^2+^ Chelating Activity

For determination of chelating activity (ChA), the extract (100 µL) and methanol solution of FeSO_4_ (100 µL, 1.58 mg/mL) were mixed for the 10 min [23]. Afterwards, ferrozine (3-(2-Pyridyl)-5,6-diphenyl-1,2,4-triazine-*p*,*p*′-disulfonic acid monosodium salt hydrate) solution (100 µL, 0.16 mg/mL) was added. Reaction mixture was incubated for 15 min in the dark and the absorbance was recorded at 562 nm. Reaction mixture containing water or ethylenediaminetetraacetic acid (EDTA) solution (100 µL), instead of the extract, served as the negative and positive control, respectively. ChA was calculated according to Equation (2):

(2) $$\mathrm{ChA}\;\left(\%\right)=\frac{A_{0}-A_{s}}{A_{0}}\times 100$$

where A_0_ is the absorbance of the negative control and As is the absorbance of the respective extract. ChA IC_50_ was calculated as the concentration of the extract which chelates 50% of Fe^2+^ present in the solution and expressed as µL of extract per mL of solution (μL extract/mL).

### 2.11. Antioxidant Activity in β-Carotene-Linoleic Acid Assay

For determination of antioxidant activity in β-carotene-linoleic acid assay (*ANTOx*) [24], 200 μL of the β-carotene (6.7 μg/mL) aqueous emulsion (containing 0.2 mg of β-carotene, 20 mg of linoleic acid and 200 mg of Tween 40 in 50 mL of aerated distilled water) was mixed in the extract solution in methanol (50 μL) and incubated at 50 °C for 60 min. Reaction mixture containing methanol or BHA solution, instead of the extract, served as the negative and positive control, respectively. The *ANTOx* was calculated based on the absorbance recorded after 0 min and 60 min as follows in Equation (3):

(3) $$\mathrm{ANTOx}\;\left(\%\right)=\frac{A_{s60}-A_{c60}}{A_{c0}-A_{s60}}\times 100$$

where A_c0_ and A_c60_ are the absorbances of the water control after 0 and 60 min, respectively, while A_a60_ is the absorbance of the sample after 60 min. ANTOx IC_50_ was calculated as the concentration of the extract that protects 50% β-carotene present in the solution after 60 min and expressed as μL of extract/mL of solution (μL extract/mL).

### 2.12. Elastase Inhibitory Activity

For elastase inhibitory activity determination [25], 100 μL of varying concentrations of extract solution in Tris-HCl buffer (0.1 M, pH 8.0) was mixed with 25 µL elastase solution (0.05 mg/mL) and left at room temperature for 5 min. Afterwards, the phosphate buffer saline solution of *N*-succinyl-(Ala)_3_-nitroanilide (SANA, 70 µL, 0.410 mg/mL) was added and the absorbance was measured at 410 nm after additional 40 min. Elastase inhibitory activity (ELAInh) was calculated using the Equation (4):

(4) $$\mathrm{ELAInh}\;\left(\%\right)=\frac{A_{0}-A_{s}}{A_{0}}\times 100$$

where A_0_ is the absorbance of the negative control and As is the absorbance of the solution containing respective extract. Ursolic acid (UA) was applied as the standard elastase inhibitor. ELA IC_50_ values (μL extract/mL) were calculated as the concentration of the extract that inhibited 50% of elastase activity.

### 2.13. Collagenase Inhibitory Activity

For collagenase inhibitory activity determination, 40 μL of varying concentrations of extract solution in Tris-HCl buffer (0.1 M, pH 7.5) was mixed with 20 µL collagenase solution (0.1 mg/mL) and left at room temperature for 5 min. After that, the gelatin solution (40 µL, 3.34 mg/mL) in the same buffer was added, and mixture was incubated for additional 40 min at 37 °C. Then, 40 µL of stop reagent (25 mM EDTA in 12% (*w*/*w*) aqueous PEG solution) and 90 µL of ninhydrin solution (0.14 M) were added to the reaction mixture and incubated for 15 min at 80 °C. After cooling down, 90 µL of citric buffer was added and the absorbance was measured at 545 nm. Reaction mixture containing buffer or gallic acid aqueous solution was used instead of extract as the negative and positive control. Collagenase inhibitory activity (COLInh) was calculated using the Equation (5):

(5) $$\mathrm{COLInh}\;\left(\%\right)=\frac{A_{0}-A_{s}}{A_{0}}\times 100$$

where A_0_ is the absorbance of the negative control and As is the absorbance of the solution containing respective extract. COLInh IC_50_ was calculated as the concentration of the extract that inhibited 50% of collagenase activity and expressed as μL of extract/mL of solution (μL extract/mL).

### 2.14. Lipoxygenase Inhibitory Activity

For lipoxygenase (LOX) inhibitory activity [26], 25 μL of LOX solution (0.0032 mg/mL), 100 μL of extract solution, and 50 μL of phosphate buffer saline (PBS, pH 8, 100 μM) were mixed. After 5 min, 50 μL of linoleic acid in phosphate buffer (pH 8, 100 μM) were mixed and incubated at 25 °C. After 45 min the absorbance was measured at 234 nm. Reaction mixture containing buffer or nordihydroguaiaretic acid (NDGA) solution instead of extract served as the negative and positive control. LOX inhibitory activity (LOXInh) was calculated as in Equation (6):

(6) $$\mathrm{LOXInh}\;\left(\%\right)=\frac{A_{0}-A_{s}}{A_{0}}\times 100$$

where A_0_ is the absorbance of the negative control and As is the absorbance of the corresponding extract. LOXInh IC_50_ was calculated as the concentration of the extract that inhibited 50% of LOX activity and expressed as μL of extract/mL of solution.

### 2.15. Hyaluronidase Inhibitory Activity

For hyaluronidase (LOX) inhibitory activity [27], 25 µL of the extract solution and 20 µL of hyaluronidase solution (4 mg/mL) was mixed and incubated for 20 min at 37 °C. After 20 min, 40 µL of 12.5 mM CaCl_2_ and incubated for additional 20 min at 37 °C. Sodium hyaluronate (50 µL, 3.5 mg/mL) was added and incubated for at 37 °C with constant shaking. After 40 min, reaction was stopped by adding 20 µL of 0.9 M NaOH and 40 µL of 0.2 M sodium tetraborate and heating for 3 min at 100 °C. Then, 160 µL of *p*-dimethylaminobenzaldehide reagent (DMABA) (0.25 g DMABA dissolved in 4.4 mL of acetic acid and 0.6 mL of 10 M HCl) was added and reaction mixture incubated at 37 °C for additional 10 min. Absorbance was measured at 585 nm. Tannic acid was used as positive control. Hyaluronidase inhibitory activity (HYALInh) was calculated as shown in Equation (7):

(7) $$\mathrm{HYALInh}\;\left(\%\right)=\frac{A_{0}-A_{s}}{A_{0}}\times 100$$

where A_0_ is the absorbance of the negative control and As is the absorbance of the corresponding extract. HYALInh IC_50_ was calculated as the concentration of the extract that inhibited 50% of hyaluronidase activity and expressed as μL of extract/mL of solution.

### 2.16. Statistical Analysis

Experimental design for 2LFD and BBD, as well as RSM, were performed by using Design Expert software version 8.0.6 (Stat-Ease, Minneapolis, MN, USA). Statistical evaluations were made applying one-way ANOVA followed by Tukey’s post-hoc test for multiple comparisons between the extracts using PrismGraphPad 8 (GraphPad Software, Inc., San Diego, USA). *p* values < 0.05 were considered statistically significant. The measurements were made in triplicate and the results presented as mean ± standard deviation. The IC_50_ values were determined using the linear regression analysis and the results obtained using seven extracts’ concentration.

## 3. Results and Discussion

### 3.1. Screening of Extraction Conditions Using 2 Level Factorial Design

Among many plant constituents, plant phenols are among the most appreciated in cosmeceutical products. They can exert beneficial effects on skin aging and age-associated skin diseases by preventing or delaying cellular senescence. For example, it has been shown that plant phenols mitigate increased levels of free-radical production and disrupt the activation of NF-κB and related pathways. They can also modulate pro-inflammatory gene expression, such as the inhibition of cyclooxygenase-2 (COX-2) or inducible nitric oxide synthase (iNO) [28]. Plant phenols have also been reported to possess substantial skin photoprotective effects [29]. Among many bioactive polyphenols in *J. montana*, especially important are flavonoid luteolin and its glycosides. It was found that luteolin derivatives display anti-melanoma activity [15], delay skin aging, enhance wound healing, and improve the skin condition in various inflammatory dermatological problems [14].

In order to determine the extraction variables that most strongly influence extraction efficacy of biologically active phenolic compounds from *J. montana*, 2LFD was used. Six independent variables selected for screening process were as follows: GL content, temperature, extraction time, lactic acid content, drug weight, and USP. GL is a common, non-toxic ingredient in cosmetic products. However, due to its high viscosity, it was not possible to use undiluted GL for the preparation of *J. montana* extracts. Therefore, its content was set between 10% (*w:w*) and 90% (*w:w*) to enable easier handling of the prepared solutions. Unlike non-toxic alcohol GL, lactic acid could have both beneficial and adverse effects depending on the concentration in the cosmetic product. Topical application of low concentrations of lactic acid may slightly disrupt the cohesion of the corneocytes in the skin barrier, and give skin a more youthful appearance, a feature widely utilized in so-called chemical peelings [21]. Still, applications of high concentrations may as well result in skin irritation [30]. To avoid possible side-effects of high lactic acid concentration, its content was set between 1% and 5% (*w:w*). Using UAE, 32 extracts were prepared, and the concentrations of target phenolics were determined (Table 1).

From the data presented in Table 1, it may be observed that TP concentration in the extracts depended greatly on the extraction parameters. For instance, TP ranged from 93.38 μg/mL in Run 11 to 626.90 μg/mL in Run 7, indicating an almost seven-fold increase in the reaction yield. The influence of the extraction parameters on the luteolin yield was even more impressive. The difference in luteolin content between Run 11 and Run 7 was around 35-fold, further stressing the importance of selection of the most successful extraction conditions for maximization of each response. To determine the statistical significance of the obtained models, *p*-values and *F*-test were applied (ANOVA, Table S1). The calculated *F*-values of both models were higher than 16, while the *p*-values for the models were lower than 0.05. This showed the significance of the selected models, as well as their suitability for the interpretation of the experimental data. The determination coefficients (*R*^2^) for TP and luteolin extraction were moderately high (0.8287 and 0.7618), showing that the observed values were reasonably well described by this model. The predicted *R*^2^ was in good agreement with the adjusted ones, additionally proving the value of the model. Temperature and drug weight were the most influential factors for TP extraction with an overall contribution of 55.23% and 18.43%, respectively (Table S2). Furthermore, glycerol content in combination with temperature also exerted influence on TP yield. The reason high temperature positively affected the TP of the extracts, especially in combination with glycerol content, is probably related to the reduction of the viscosity of organic solvents at higher temperatures, which is especially important in cases of highly viscous solvents such as glycerol [4]. The relative importance of the drug weight indicates that the saturation of TP was not achieved in all extraction conditions. On the other hand, the most influential factors for luteolin extraction, as stated in Table S2, are USP (56.85%), a combination of glycerol content×USP (10.56%), and a combination of drug weight×USP (5.69%). The importance of USP in luteolin extraction may be explained by fragmentation of the cell wall caused by the increased ultrasound-waves power which allows an easier approach of the solvent to the cell and increases the mass transfer. Such a clear relationship between USP and the TP might be missing because various phenolic structures are included in TP. Each of them has a different stability and some might be susceptible to oxidation or hydrolysis when exposed to ultrasound [4]. Time and lactic acid content did not significantly affect the extraction efficiency.

### 3.2. Optimization of Selected Extraction Conditions Using Box-Behnken Design

In order to find the best conditions that allow for the extraction of the most bioactive compounds, BBD was employed, followed by extraction optimization using RSM. Using the 2LFD results, four independent variables that exerted the most influence on the TP and LUT extraction (glycerol content, temperature, plant material weight, and USP) were chosen for further optimization. In addition to the TP and luteolin content, DPPH RSA of the extracts was also assessed as a simple tool to evaluate antioxidant activity of the extracts and use the results to prepare the extracts with strong antiradical properties. Extraction time was set to 20 min, while lactic acid, the variable that did not statistically significantly influence the extraction efficiency in 2LFD, was not added. Using the extraction conditions presented in Table 2, 29 extracts were prepared and analyzed.

The amount of extracted TP differed among extracts with Run 16 containing 208.05 μg/mL and Run 28 containing 742.10 μg/mL TP, indicating a 3.57-fold increase in TP yield. The difference ratio between the extraction in the most and least successful runs (Run 16 and 7) for luteolin was even higher (17.95-fold). The best RSA IC_50_ value (5.00 μL extract/mL) was obtained for Run 11, while the highest RSA IC_50_ value was displayed by Run 29 (23.32 μL extract/mL). RSA showed a similar ratio as TP with the difference among Run 11 and Run 29 was set to 4.66. Equations (8)–(10) show the fitted model equations with statistically significant factors (*p* < 0.05) marked with asterisk (*).

TP (μg/mL) = −197.10 × X_7_^2^ (*) − 21.95 × X_8_^2^ − 66.49 × X_9_^2^ (*) − 38.71 × X_10_^2^ + 49.06 × X_7_ × X_8_ + 50.44 × X_7_ × X_9_ − 48.93 × X_7_ × X_10_ − 13.10 × X_8_ × X_9_ − 16.38 × X_8_ × X_10_ − 1.08 × X_9_ × X_10_ + 36.91 × X_7_ + 25.41 × X_8_ (*) + 140.26 × X_9_ − 66.73 × X_10_ + 635.87 (8)

Luteolin (μg/mL) = −102.02 × X_7_^2^ (*) − 50.38 × X_8_^2^ (*) − 29.93 × X_9_^2^ (*) − 25.57 × X_10_^2^ (*) + 0.22 × X_7_ × X_8_ + 4.41 × X_7_ × X_9_ − 4.14 × X_7_ × X_10_ + 2.07 × X_8_ × X_9_ − 15.15 × X_8_ × X_10_ − 9.13 × X_9_ × X_10_ + 3.98 × X_7_ − 22.31 × X_8_ (*) + 9.83 × X_9_ − 2.54 × X_10_ + 160.07 (9)

RSA IC_50_ (μL extract/mL) = 5.27 × X_7_^2^ (*) + 0.75 × X_8_^2^ + 1.98 × X_9_^2^ (*) − 1.93 × X_10_^2^ − 1.00 × X_7_ × X_8_ + 1.81 × X_7_ × X_9_ − 0.47 × X_7_ × X_10_ + 1.66 × X_8_ × X_9_ − 1.45 × X_8_ × X_10_ + 0.81 × X_9_ × X_10_ − 4.97 × X_7_ (*) − 3.26 × X_8_ (*) − 0.58 × X_9_ + 0.01 × X_10_ + 8.75 (10)

In general, all the extractions were affected by GL content and plant material weight as quadratic terms, either negative (TP and luteolin) or positive (RSA IC_50_) (Equations (8)–(10)). This indicates that for each dependent variable there is a value of glycerol content and plant material weight that is best suited for the achievement of the extraction goals. In the case of glycerol content, it is due to the optimal polarity of the extraction solvent that is best suited for the extraction of the target compounds. It is interesting to note that the significance of plant material weight as quadratic term means that the increase of this variable may negatively influence TP and luteolin concentration, as well as lead to preparation of the extract with lower antiradical activity. Dry plant material may re-hydrate in water/glycerol solution. Thus, the swelling of the material increases the glycerol content, thus changing the composition and polarity of the solvent. This effect is more pronounced with higher amounts of herbal material. Furthermore, the higher temperature as linear term positively affected TP extraction efficiency and negatively affected the RSA IC_50_ of the extracts. In addition to faster matter and energy transfer, this effect may be associated with a decrease in the viscosity of the glycerol solution at higher temperatures, e.g., the viscosity of pure glycerol decreases considerably by increasing the temperature from 30 °C to 80 °C [31,32], and such improved hydrodynamic conditions positively affect the extraction efficiency. However, the luteolin concentration was negatively affected by high temperature as both quadratic and linear term indicating that this flavonoid has limited stability at high temperatures. Luteolin extraction was affected by USP as negative quadratic term indicating that medium-strength USP is best suited for luteolin extraction.

Statistical significance of the proposed models was evaluated using ANOVA (Tables S3–S5). The analysis of variance showed relatively high *F*-values (>9) in combination with very low *p*-values (<0.01) which indicates that they appropriately represented the correlation between the independent and dependent variables. Moreover, the insignificant values of lack-of-fit tests (*p* > 0.05) proved that the models were acceptable to describe the obtained experimental data. The relatively high determination coefficients (*R*^2^ > 0.90) with relatively small difference between the adjusted and predicted determination coefficients shows good agreement of the experimental values and those predicted by the chosen models.

The values of independent variables best fitted for the preparation of extracts with the desired qualities were calculated. Those conditions were used to prepare optimized extracts having the highest TP, luteolin content, and lowest RSA IC_50_ (Table 3). The predicted values of the responses in the prepared extracts were in good accordance with the predicted ones with response deviation of less than ± 5%. The obtainer results further stress the ability of selected models to adequately describe and predict values for each response.

### 3.3. Comparison of Optimized and Deep Eutectic Solvents Extracts

NADES are known as green and are efficient tools for green extraction of polyphenols from various sources [33]. Among the NADES applications, the extraction of phenolic compounds and preparation of the extracts to be used in cosmeceutical products takes a prominent place. Additionally, they can be used in cosmeceutical products without further purification steps, due to the non-toxic and natural structure of their components [34]. In addition to their non-toxicity, NADES-based extracts are promising carriers in new drug delivery systems for topical applications since they can affect the permeation of active molecules [35].

To compare the efficacy of GL- and NADES-based extraction, a series of NADES, previously described in the literature as suitable for the extraction of natural phenolic compounds, were used. Generally, they consisted of affordable, readily available components commonly used for skin hydration and/or conditioning in cosmetic products, such as Bet, GL, Glu, urea, and Pro [9,20,36]. The conditions for the extract preparations were identical to those used for the preparation of optimized extracts, with the sole difference being the use of NADES instead of GL/water mixtures. The abbreviated names of the NADES-based extracts were created from the abbreviation of the corresponding NADES (given in Section 2.6) that was used for extraction instead of GL/water mixtures, the number indicating the percentage of NADES in water, and suffixes (-TP, -LUT or -RSA) indicating the conditions applied for extracts’ preparation (Table 3). For example, PG-50-LUT is the extract prepared using PG (Pro:GL, 2:5, *w:w*) NADES instead of GL/water mixture, diluted with water in 1:1 ratio, prepared as described in Table 3 for OPT-LUT (at temperature 56 °C, using 0.47 g of plant material and USP of 576 W). 

**Figure 1:**
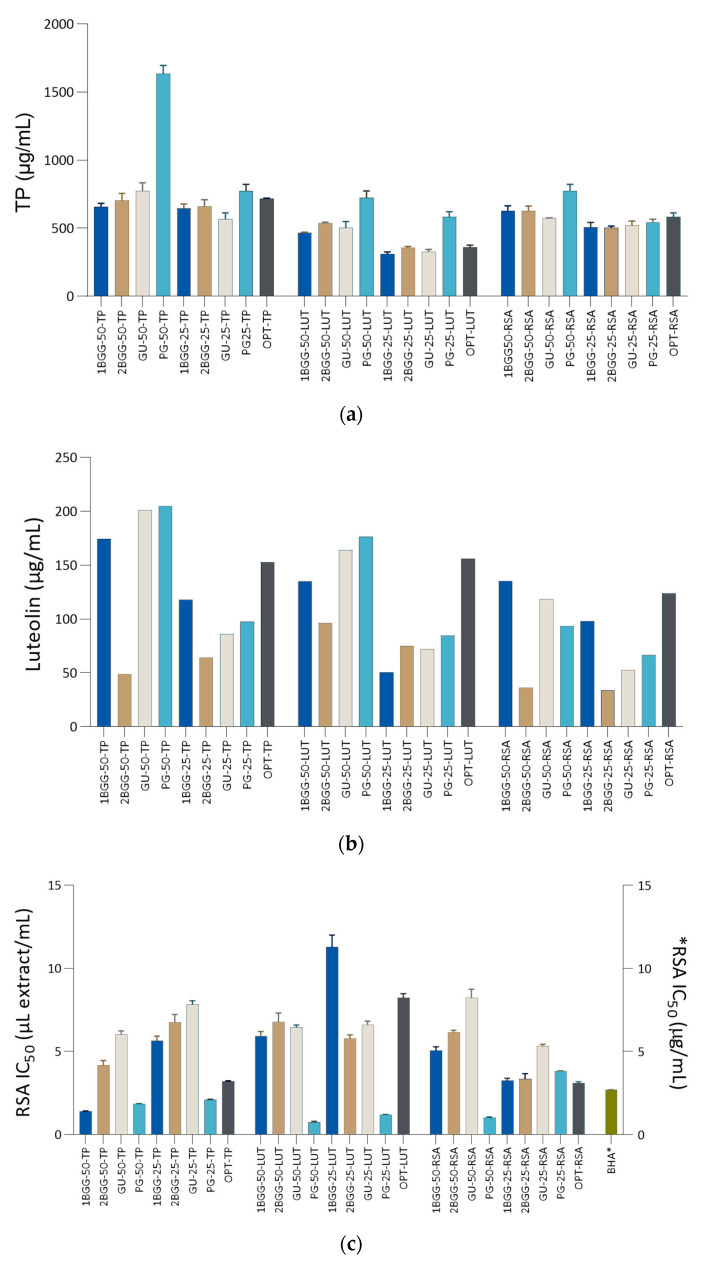
Comparison of NADES and optimal extracts based on responses: (**a**) total phenolic content (TP), (**b**) luteolin content, and (**c**) radical scavenging activity (RSA). Values in (**a**,**c**) are average of three replications ± SD. BHA = butylated hydroxyanisole. Asterisk indicates that the unit is placed at the right ordinate. Abbreviated names for NADES-based extracts are explained in Section 3.3.

The prepared NADES extracts were compared with optimized GL-based extracts to compare the efficiency of two green extraction methods. Figure 1a shows the TP of NADES-based and optimized extracts. A detailed statistical comparison using ANOVA may be found in Table S6. As it may be seen in Figure 1a and Table S6, the extracts prepared using OPT-TP conditions contained the most TP. For example, OPT-TP had significantly more TP than OPT-LUT and OPT-RSA, which was in line with the model prediction. However, statistically, the TP content of OPT-TP extract values was in the middle, with a series of statistically non-distinctive NADES-based extracts (Table S6). In most cases, the selection of NADES solvent did not play a significant role when it came to TP yield. However, one of the extracts among those shown in Figure 1a had dramatically higher TP content than the others. It was PG-50-TP that had more than 2-fold higher TP content than either OPT-TP or the next best extract, GU-50-TP. 

In order to establish if the solution with such high TP yield had reached its saturation, two more extracts were prepared using the PG-50 solvent and the same extraction conditions, with the sole exception being the weight of plant material used in the extraction. Thus, for the preparation of PG-50-TP-0.8 and PG-50-TP-1.0, 0.8 g and 1.0 g of plant material was used, respectively. Those two extracts contained even higher TP content than PG-50-TP, indicating superiority of PG-50 as a solvent for the extraction of TP from *J. montana*. In general, NADES-50 solvents contained more phenols than the analogue NADES-25 solvents, but the difference was not always statistically significant. As it may be observed in Figure 1b and Table S6, PG-50-TP was the extract with the highest luteolin content, followed closely by GU-50-TP that contained an only slightly lower concentration of that flavonoid. OPT-LUT, the extract that was optimized to the maximal luteolin concentration, expectedly contained more luteolin than OPT-TP and OPT RSA. However, it also contained 31.1% less luteolin than the best NADES-based extract, PG-50-TP. Several NADES-based solvents prepared using the TP-optimization procedure (e.g., GU-50-TP and PG-50-TP) contained more luteolin than either OPT-LUT or their -LUT counterparts. This is partly expected because of the higher plant material weight used for their preparation (0.6 g vs. 0.47 g, Table 3). It seems that saturation of those extracts with luteolin is achieved at higher luteolin concentrations than in 50% GL, used for OPT-LUT preparation. This hypothesis was confirmed by analyzing luteolin concentration in PG-50-TP-0.8 and PG-50-TP-1.0 (Table S6). Expectedly, both extracts contained more luteolin than PG-50-TP. The selection of NADES for luteolin extraction was very important as, in general, the difference between luteolin yield between the most and the least successful NADES for each set of extraction conditions was approximately 2-fold. As luteolin is a moderately polar flavonoid, NADES-50 solvents were more successful in its extraction than the analogue NADES-25 solvents.

Antiradical activity of the extracts is displayed in Figure 1c and Table S6. The positive control for RSA (BHA) was also evaluated and displayed. It is important to note that the activity of the extracts may not be directly compared to BHA because their activity is expressed in different measurement units (μL extract/mL and μg/mL, for the extracts and, respectively). However, for comparison purposes, it is possible to regard the activity of BHA as volume equivalents of 1 mg/mL solutions. The NADES PG-50 again yielded the extract with the most desired characteristics, as PG-50-LUT was the extract with the most pronounced antiradical characteristics, closely followed by PG-50-RSA and PG-25-LUT. PG-50-TP, the extract with the highest amount of TP, also displayed notable antiradical activity. Expectedly, as they contained more phenolic antioxidants, PG-50-TP-0.8 and PG-50-TP-1.0 displayed even stronger antiradical activity than PG-50-TP. All PG-based extracts had an RSA IC_50_ value similar or even lower than the BHA solution. NADES-50 extracts prepared based on OPT-TP and OPT-LUT procedures (Table 3) were, in general, better radical scavengers than analogue NADES-25 extracts. Interestingly, the opposite was true for the extracts prepared based on OPT-RSA procedure. In general, selection of NADES had a great impact on the RSA IC_50_ of the extracts. Even though 1BGG-25-LUT and PG-25-LUT were prepared using the same conditions, RSA IC_50_ of the former was more than 9-fold higher than the latter.

### 3.4. Antioxidant Activity

Botanical ingredients represent one of the largest categories of natural active substances used in dermatology. Besides simple hydration and antioxidant protection, botanical ingredients often display other biological properties beneficial for skin. One of the most important characteristics of botanical ingredients is their antioxidant activity, and thus the ability to counteract oxidative stress. Increased oxidative stress gradually increases the activity of extracellular matrix enzymes and causes DNA damage that leads to various issues such as genetic, hormonal, and metabolic changes [37,38]. By interfering with the proteolytic enzymes that accelerate the aging process and degradation of the skin, antioxidants prevent skin changes caused by aging and, consequently, positively affect the appearance of the skin [21]. In this work, the antioxidant activity of GL-based optimized extracts (OPT-TP, OPT-LUT and OPT-RSA) was analyzed. As the extract containing proline-based NADES, PG50-TP, had the best general performances with the best TP and luteolin and relatively low RSA IC_50_ value, its activity was also determined and compared to the GL-based solvents. The antioxidant activity of the extracts prepared was assessed using three methods: the influence of the prepared extracts on the free radicals (as modeled by DPPH free radical), chelating activity on Fe^2+^ ions, and the activity in β-carotene-linoleic acid system. These methods represent a quick, dependable, and affordable way for the determination of in vitro antioxidant properties of natural substances in extracts. As already described in the investigation of RSA, positive controls were used for general comparison purposes. 

**Figure 2:**
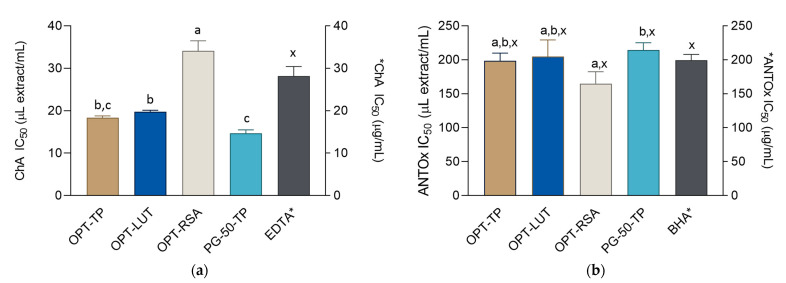
Antioxidant activity of the extracts and positive controls EDTA (ethylenediaminetetraacetic acid) and BHA (butylated hydroxyanisole) in: (**a**) chelating activity (ChA) and (**b**) β-carotene-linoleate assay. The activities are shown as IC_50_ values ± SD. ^a–c^ = differences between the extracts within a column (Tukey post-test, *p* < 0.05). ^x^ = differences with the positive control (Dunnett’s post-test, *p* < 0.05). Columns not connected with the same letter are statistically different. Asterisk indicates that the unit is placed at the right ordinate.

Radical scavengers offer protection against oxidative damage of skin macromolecules associated with the effects of free radicals and UV radiation on the skin [39]. A stable DPPH-free radical is often used to determine radical scavenging activity of natural compounds. In its radical form, DPPH shows an absorbance peak at 517 nm. However, in reaction with an antioxidant, the absorption of the solution decreases due to the formation of the DPPH non-radical form [40]. Antiradical properties of all the prepared extracts is displayed in the previous section (Figure 2c, Table S6). Among the extracts included in further studies of biological activity, PG-50-TP displayed the strongest antiradical activity, statistically significantly stronger than the activity of the BHA solution. It was closely followed by OPT-TP and OPT-RSA, the extracts that displayed an activity equal to the control BHA solution. High antiradical activity of OPT-TP is not surprising because phenolics, due to their chemical structures, may donate hydrogen to free radicals and thus strongly contribute to the antioxidant activity of the prepared extracts [41]. A previous study has shown that luteolin-rich *J. montana* extracts are potent radical scavengers [18]; however, it is difficult to compare the results of that study to those obtained in this work due to different measurement units. In the same work, performed on a smaller number of extracts, it was reported that the RSA correlates with luteolin content [18]; however, the results obtained in this study indicate that other phenolics present in the extracts also play an important part in the RSA of the extracts.

Due to the pro-oxidant nature of Iron [42], high levels of this transition metal may impair the stability of the cosmetic product and, as a result, shorten its shelf-life [43]. Furthermore, it has recently been shown that the exposure of the skin to UV radiation leads to an increase in cutaneous intracellular catalytic iron levels and, subsequently, to the generation of free radicals. By binding the free iron, metal chelators may thus prevent UV-induced photodamage to the skin and consequently inhibit photoaging [44]. Chelating activity of the extracts was investigated using assay based on the ability of ferrozine to form chromophore with iron cations (Fe^2+^) with a strong absorbance at 562 nm. As iron chelating agents lower the concentration of Fe^2+^ in the solution, the concentration of the ferrozine-iron complex also decreases, resulting in a decline in absorbance at 562 nm [45]. The results of the chelating ability for the extracts and positive control, EDTA, are presented in Figure 2a. The obtained IC_50_ values showed that all extracts, except OPT-RSA, were better chelating agents than the 1 mg/mL EDTA solution, indicating that they were able to chelate Fe^2+^ ions of transition metals and thus retard the oxidation processes. There were no statistically significant differences between OPT-LUT and OPT-TP. However, PG-50-TP was a significantly stronger chelating agent. Flavonoids are well-known chelating agents able to bind transition ions at different pH values. For example, luteolin forms stable complexes with metal cations that are not prone to oxidative degradation. This is a clear advantage over quercetin, another flavonoid widely distributed in the plant kingdom, that, in addition to complexation, is also susceptible to oxidation that occurs via both oxygenation and hydroxylation mechanisms [46]. Other studies have shown that various phenolics, including luteolin derivatives, play an important role not only in chelating activity, but also in the overall antioxidant protective activity of herbal extracts, including sage (*Salvia officinalis*) [47].

In addition to protecting skin macromolecules from exo- and endogenous free radicals and harmful solar radiation [29], antioxidant agents are indispensable for the proper storage of cosmetic products because they protect their ingredients against oxidative influences from the environment. Especially important from the preservative point of view is the protection against peroxidation of unsaturated fatty acids that are common ingredients in creams and lotions [48]. The β-carotene linoleic acid assay gives an insight into the behavior of the extracts in the mixtures with unsaturated fatty acids. The linoleic acid-free radical, formed upon removal of a hydrogen atom located between two double bonds of linoleic acid, reacts with β-carotene [24]. The consequence is loss of conjugation and, accordingly, a decrease in absorbance at 470 nm. Antioxidants present in the solution can prevent the degradation of β-carotene by reacting with the linoleate-free radical or any other radical formed in the solution. All the tested extracts displayed the activity statistically equal to BHA, indicating excellent antioxidant properties in this assay. OPT-RSA, the extract optimized to display the strongest antiradical properties, was the most active in this assay, which is in accordance with the radical-based mechanism of the assay. It is well known that phenolics-rich plant extracts are good scavengers of free-radicals in this assay, and the extracts containing luteolin derivatives are no exception. The examples include the extracts of *Thymus serpyllum* [49] and *Hyacinthoides lingulata* [50].

### 3.5. Enzyme Inhibiting Activity

The expected activity of the plant extracts in cosmeceutical products extends beyond simple hydration and antioxidant protection. They may act as functional ingredients and delay or prevent processes that negatively influence skin health and appearance, for example, by protecting the skin polysaccharides or proteins against enzymatic degradation induced by exposure to UV radiation or other environmental stressors. Herbal-based formulations can reduce or slow down the aging process of the skin by acting as inhibitors of enzymes involved in the cellular aging process, such as elastase, collagenase, and hyaluronidase. Their excessive activity can cause a premature breakdown of elastin, collagen, and hyaluronic acid, which accelerates the skin aging process and causes aesthetically visible effects such as reduced elasticity and skin tone, the appearance of wrinkles, and dehydrated skin [51]. For example, clinical trials confirm that the inhibition of elastase activity indicates the important anti-aging potential of the natural product and other compounds that display it [52]. In addition, tissue inflammation is a significant characteristic of the ageing process in the skin and other organs. Plant metabolites may inhibit the development of inflammation-induced skin changes and thus contribute to the anti-ageing activity of the product [53]. In this study, anti- elastase, -collagenase, -lipoxygenase, and -hyaluronidase activity was investigated. As described in the previous subsection, positive controls were tested for general comparison purposes, as it is possible to regard their activity as volume equivalents of the 1 mg/mL solutions. 

**Figure 3:**
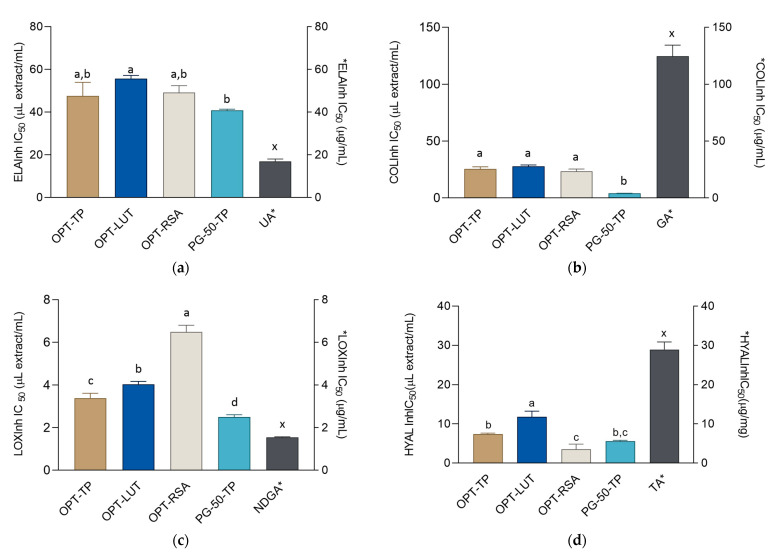
Inhibitory activity of the extracts and corresponding positive controls: ursolic acid (UA), gallic acid (GA), nordihydroguaiaretic acid (NDGA), and tannic acid (TA) on: (**a**) elastase, (**b**) collagenase, (**c**) lipoxygenase, and (**d**) hyaluronidase. The activities are shown as IC_50_ values ± SD. ^a–d^ = differences between the extracts within a column (Tukey post-test, *p* < 0.05). ^x^ = differences with the positive control (Dunnett’s post-test, *p* < 0.05). Columns not connected with the same letter are statistically different. Asterisk indicates that the unit is placed at the right ordinate.

Elastin is an important protein responsible for maintaining the mechanical properties of the skin [54]. The degradation of elastin is induced by activity of the enzyme elastase, which is directly related to skin aging and oxidative stress [55]. The elastase inhibitory activity assay of extracts, along with the UA positive control, is shown in Figure 3a. The extracts’ IC_50_ values were somewhat lower than the activity of the positive control. Again, PG-50-TP was the most successful elastase inhibitor. Collagen is the dominant component of skin cell tissue and is responsible for the strength and stability of skin cell tissue. Collagenase is an enzyme active in the extracellular matrix that contributes to the degradation of collagen. With aging and various external influences (UV radiation), its activity increases and leads to the formation of wrinkles and loss of skin tone [56]. The collagenase-inhibitory effect of the extracts is shown in Figure 3b. The extracts were stronger collagenase inhibitors than the positive control, gallic acid. The eutectic-based extract (PG-50-TP) demonstrated a statistically significant superior inhibitory effect with an IC_50_ value of 4.14 ± 0.02 µL of extract/mL. This confirms the results of a previous study which found that luteolin-rich *J. montana* extracts are excellent inhibitors of elastase [18].

The anti-inflammatory effect on the skin is considered to be one of the most important areas for research on herbal extracts. Inflammatory skin reactions that cause redness, rash, edema, or defective physiological function of the skin are becoming more frequent [57]. The isoenzyme lipoxygenase found in the skin has a role in protecting the skin’s cell barrier, inflammatory skin processes, wound healing, and modulating epithelial proliferation and differentiation [58]. The results of the LOX inhibition assay were presented in Figure 3c. The extracts demonstrated comparable but somewhat lower activity than the positive control. Again, PG-50-TP demonstrated the most pronounced inhibitory effect, 36.1% higher that OPT-TP as the second-best extract. The activity of PG-50-TP was comparable with the NDGA positive control, confirming a potent inhibitory effect of this extract. The reduction of the skin hydration layer leads to a reduction of turgor, resilience, pliability, and premature skin aging. Hyaluronic acid, a polysaccharide with a unique capacity to retain water, is one of the key molecules involved in skin hydration [59]. However, in skin aging and various pathological processes, hyaluronic acid is gradually degraded by hyaluronidase, the key enzyme that controls the turnover of hyaluronic acid in human skin [60]. Thus, inhibition of hyaluronidase leads to retention of skin moisture and is one of the promising approaches for the maintenance of a youthful skin appearance. As it may be observed in Figure 3d, all the tested extracts were excellent hyaluronidase inhibitors, with their activity being better than the activity of the positive control, tannic acid. Similar to the results of the previous experiments, PG-50-TP was the most active, displaying the most pronounced anti-hyaluronidase activity, closely followed by OPT-RSA. Various flavonoid derivatives, including luteolin 7-*O*-glucoside, are good inhibitors of collagenase, elastase, and hyaluronidase, the enzymes whose activity may have deteriorating consequences on skin [61]. For example, bioassay-guided fractionation and isolation has exposed luteolin-7-*O*-glucoside as the main active component of the aerial parts of *Daphne oleoides*, capable not only for the inhibition of hyaluronidase and collagenase activity, but also interfering with the inflammatory processes [62]. Thus, it is reasonable to expect that luteolin-7-*O*-glucoside present in *J. montana* plays an equally important role in the extracts prepared in this work.

## 4. Conclusions

GL and NADES were found to be suitable solvents for the UAE extraction of phenolic constituents from *J. montana*. Among the NADES solvents, PG-50 was far superior for the extraction of luteolin and other phenolic compounds, confirming the importance of careful selection of solvent components in NADES-based extraction. The optimized glycerolic extracts, OPT-TP, OPT-LUT, and OPT-RSA, as well as the most efficient NADES-based extract, PG-50-TP, were excellent antioxidants and Fe^2+^ ion chelators. Furthermore, they were able to impair elastase and LOX activity. In addition to that, they were potent inhibitors of collagenase and hyaluronidase. The observed antioxidant- and enzyme-inhibiting activity may add additional beneficial effects to the products containing *J. montana* extracts and make them promising constituents of specialized cosmeceutical formulations. The fact that they were prepared using green methods and environmentally friendly and non-toxic solvents further increases the value of the prepared extracts. Among the tested extracts, PG-50-TP displayed the best activity in a majority of performed assays, assuring the status of the best candidate for cosmeceutical product development.

## Figures and Tables

**Table 1:** Independent variables in the 2-Level Factorial design and content of total phenols (TP) and luteolin (LUT) in the extracts.

Standard	Run	X_1_	X_2_	X_3_	X_4_	X_5_	X_6_	TP	LUT
		(%, *w:w*)	(°C)	(min)	(%, *w:w*)	(g)	(W)	(μg/mL)	(μg/mL)
22	1	90	30	30	0	0.4	720	172.84	32.54
29	2	10	30	30	5	0.4	720	154.33	20.92
2	3	90	30	10	0	0.2	720	95.94	19.80
30	4	90	30	30	5	0.4	144	179.22	1.42
15	5	10	80	30	5	0.2	720	309.41	7.66
24	6	90	80	30	0	0.4	144	614.46	3.08
32	7	90	80	30	5	0.4	720	626.90	44.38
28	8	90	80	10	5	0.4	144	412.47	1.91
5	9	10	30	30	0	0.2	720	193.26	14.05
27	10	10	80	10	5	0.4	720	502.14	15.94
10	11	90	30	10	5	0.2	144	93.38	1.26
8	12	90	80	30	0	0.2	720	331.74	18.32
6	13	90	30	30	0	0.2	144	153.37	1.89
17	14	10	30	10	0	0.4	720	337.49	8.33
20	15	90	80	10	0	0.4	720	450.45	32.97
13	16	10	30	30	5	0.2	144	285.48	1.92
26	17	90	30	10	5	0.4	720	151.78	21.82
23	18	10	80	30	0	0.4	720	468.95	17.14
19	19	10	80	10	0	0.4	144	503.73	1.77
9	20	10	30	10	5	0.2	720	213.68	15.24
12	21	90	80	10	5	0.2	720	338.76	21.86
16	22	90	80	30	5	0.2	144	354.08	13.40
11	23	10	80	10	5	0.2	144	359.82	15.20
4	24	90	80	10	0	0.2	144	344.51	2.38
14	25	90	30	30	5	0.2	720	135.82	11.27
18	26	90	30	10	0	0.4	144	168.37	1.96
1	27	10	30	10	0	0.2	144	196.77	8.20
3	28	10	80	10	0	0.2	720	258.35	6.49
25	29	10	30	10	5	0.4	144	408.96	4.92
21	30	10	30	30	0	0.4	144	328.55	10.49
7	31	10	80	30	0	0.2	144	332.70	1.98
31	32	10	80	30	5	0.4	144	506.61	3.38

X_1_ = glycerol content, X_2_ = temperature, X_3_ = extraction time, X_4_ = lactic acid content, X_5_ = drug weight, and X_6_ = ultrasound power, TP = total phenolic content.

**Table 2:** Content of TP, luteolin, and radical scavenging activity of the extracts prepared according to the Box Behnken design.

Standard	Run	X_7_	X_8_	X_9_	X_10_	TP	Luteolin	RSA IC_50_
		(%, *w:w*)	(°C)	(g)	(W)	(μg/mL)	(μg/mL)	(μL Extract/mL)
28	1	50	60	0.45	576	627.26	128.67	11.70
7	2	50	60	0.30	720	362.67	109.64	6.90
4	3	90	80	0.45	576	560.19	15.22	5.94
20	4	90	60	0.60	576	575.87	35.92	12.01
22	5	50	80	0.45	432	601.90	55.02	5.58
21	6	50	40	0.45	432	601.86	100.17	6.95
29	7	50	60	0.45	576	658.16	173.29	8.20
19	8	10	60	0.60	576	395.13	24.41	19.30
12	9	90	60	0.45	720	315.35	36.33	6.53
9	10	10	60	0.45	432	409.14	17.95	16.01
24	11	50	80	0.45	720	475.30	24.41	5.00
14	12	50	80	0.30	576	434.33	32.87	7.43
1	13	10	40	0.45	576	389.79	16.27	22.58
5	14	50	60	0.30	432	476.26	102.86	12.33
2	15	90	40	0.45	576	336.24	15.18	16.71
18	16	90	60	0.30	576	208.05	9.66	8.78
26	17	50	60	0.45	576	625.32	168.65	11.45
25	18	50	60	0.45	576	588.29	168.44	6.46
23	19	50	40	0.45	720	540.79	130.17	12.18
3	20	10	80	0.45	576	417.51	15.44	15.80
13	21	50	40	0.30	576	348.80	95.03	17.29
10	22	90	60	0.45	432	604.02	48.07	5.93
27	23	50	60	0.45	576	680.32	161.28	5.92
6	24	50	60	0.60	432	719.08	133.45	10.07
8	25	50	60	0.60	720	601.16	103.72	7.87
11	26	10	60	0.45	720	316.18	22.79	18.50
15	27	50	40	0.60	576	708.97	120.12	11.52
16	28	50	80	0.60	576	742.10	66.24	8.32
17	29	10	60	0.30	576	229.08	15.79	23.32

X_7_ = glycerol content, X_8_ = temperature, X_9_ = drug weight, and X_10_ = ultrasonication power. TP = total phenolic content, RSA = radical scavenging activity.

**Table 3:** Predicted and observed response values for the extracts prepared at optimal conditions.

Extract Name	Optimized Response	Response Aim	X_7_ (%, *w:w*)	X_8_ (°C)	X_9_ (g)	X_10_ (W)	Predicted Response Value	Observed Response Value	RD (%)
OPT-TP	TP (μg/mL)	maximized	60	70	0.60	432	775.48	740.00 ± 5.70	4.79
OPT-LUT	Luteolin (μg/mL)	maximized	50	56	0.47	576	163.24	156.23	4.48
OPT-RSA	RSA (μL extract/mL)	minimized	80	70	0.38	720	3.04	3.10 ± 0.08	−2.16

X_7_ = glycerol content, X_8_ = temperature, X_9_ = drug weight, and X_10_ = ultrasound power. TP = total phenolic content, RSA = radical scavenging activity, RD = response deviation, calculated as (Observed-Predicted)/Predicted × 100.

## Data Availability

The data presented in this study are available in article and Supplementary Material.

## Supplementary Materials

The following supporting information can be downloaded at: https://www.mdpi.com/article/10.3390/metabo13010032/s1, Table S1. Analysis of variance (ANOVA) for the 2-Level Factorial Design model. Table S2. Influence of statistically significant independent variables on total phenol and luteolin extraction in 2-Level Factorial Design. Table S3. Analysis of variance (ANOVA) for the Box-Behnken design model for TP extraction. Table S4. Analysis of variance (ANOVA) for the Box-Behnken design model for luteolin extraction. Table S5. Analysis of variance (ANOVA) for the Box-Behnken design model for RSA extraction. Table S6. Comparison of NADES and optimal extracts responses.
