# Supplementary material for: Glycerol and Natural Deep Eutectic Solvents Extraction for Preparation of Luteolin-Rich Jasione montana Extracts with Cosmeceutical Activity
Source: Metabolites. 2022 Dec 24;13(1):32. doi: 10.3390/metabo13010032 (PMC9861245; doi:10.3390/metabo13010032)
Supplement: Supplementary file 1 [file metabolites-13-00032-s001.zip › metabolites-2105064-supplementary.pdf]

metabolites MDPI

Table S1. Analysis of variance (ANOVA) for the selected 2-level factorial design model including statistically significant and hierarchical factors.

	TP				
R^2	$R^2 = 0.8287$, $R^2_P = 0.8034$, $R^2_A = 0.7594$				
Source	SS	DF	MS	*F* Value	*P*-value
Model	556700	4	139200	32.67	< 0.0001
X_1	16934.35	1	16934	3.97	0.0564
X_2	371100	1	371100	87.09	< 0.0001
X_5	123800	1	123800	29.05	< 0.0001
$X_1 \times X_2$	44959.15	1	44959	10.55	0.0031
Residual	115000	27	4260		
Cor Total	671800	31			
	**Luteolin**				
R^2	$R^2 = 0.7618$, $R^2_P = 0.7159$, $R^2_A = 0.6391$				
Source	SS	DF	MS	*F* Value	*P*-value
Model	55.76	5	11.2	16.63	< 0.0001
X_1	1.27	1	1.27	2.03	0.1666
X_5	1.02	1	1.02	1.63	0.2133
X_6	38.81	1	38.81	62.13	< 0.0001
$X_1 \times X_6$	7.30	1	7.30	11.69	0.0021
$X_5 \times X_6$	4.03	1	4.03	6.45	0.0174
Residual	16.24	26	0.62		
Cor Total	68.66	31			

X_1 = glycerol content, X_2 = temperature, X_5 = drug weight, X_6 = ultrasound power, SS = sum of squares, DF = degrees of freedom, MS = mean square, R^2_A = adjusted R^2; R^2_P = predicted R^2, and TP = total phenolic content.

Table S2. Influence of statistically significant independent variables on total phenol and luteolin extraction in 2-level factorial design.

Response	Independent variables	SS	SE	Contribution (%)
TP	X_2	371057	215.36	55.24
	X_5	123773	124.39	18.42
	$X_1 \times X_2$	44959	74.97	6.69
Luteolin	X_6	38.81	2.20	56.52
	$X_1 \times X_6$	7.30	0.96	10.63
	$X_5 \times X_6$	4.03	0.71	5.87

X_1 = glycerol content, X_2 = temperature, X_5 = drug weight, X_6 = ultrasound power, SS = sum of squares, SE = standardized effect, and TP = total phenolic content.

Table S3. Analysis of variance (ANOVA) for the Box-Behnken design model for TP extraction.

	TP				
R^2	$R^2 = 0.9472$, $R^2_P = 0.8943$, $R^2_A = 0.7279$				
Source	SS	DF	MS	*F* Value	*P*-value
Model	605200	14	43231.97	17.92	< 0.0001
Lack of Fit	28847.88	10	2884.79	2.34	0.2136
Pure Error	4921.74	4	1230.44		
X_7	16344.89	1	16344.89	6.78	0.0209

X_8	7745.77	1	7745.77	3.21	0.0948
X_9	236077.79	1	236077.79	97.87	< 0.0001
X_{10}	53440.44	1	53440.44	22.16	0.0003
$X_7 \times X_8$	9626.91	1	9626.91	3.99	0.0656
$X_7 \times X_9$	10178.42	1	10178.42	4.22	0.0591
$X_7 \times X_{10}$	9575.74	1	9575.74	3.97	0.0662
$X_8 \times X_9$	686.60	1	686.60	0.28	0.6020
$X_8 \times X_{10}$	1073.71	1	1073.71	0.45	0.5155
$X_9 \times X_{10}$	4.65	1	4.65	0.00	0.9656
X_7^2	252000.36	1	252000.36	104.47	< 0.0001
X_8^2	3126.09	1	3126.09	1.30	0.2741
X_9^2	28673.90	1	28673.90	11.89	0.0039
X_{10}^2	9719.97	1	9719.97	4.03	0.0644
Residual	33769.62	14	2412.12		
Cor Total	639017.21	28			

X_7 = glycerol content, X_8 = temperature, X_9 = drug weight, X_{10} = ultrasonication power, SS = sum of squares, DF = degrees of freedom, MS = mean square, R^2_A = adjusted R^2, R^2_P = predicted R^2, and TP = total phenolic content.

Table S4. Analysis of variance (ANOVA) for the Box-Behnken design model for luteolin extraction.

	**Luteolin**				
R^2	$R^2 = 0.9351$, $R^2_P = 0.8702$, $R^2_A = 0.6879$				
Source	SS	DF	MS	*F* Value	*P*-value
Model	83047	14	5931.93	14.41	< 0.0001
Lack of Fit	4458.13	10	445.81	1.37	0.4097
Pure Error	1306.05	4	326.51		
X_7	189.83	1	189.83	0.46	0.5082
X_8	5973.21	1	5973.21	14.51	0.0019
X_9	1160.37	1	1160.37	2.82	0.1154
X_{10}	77.40	1	77.40	0.19	0.6712
$X_7 \times X_8$	0.19	1	0.19	0.00	0.9833
$X_7 \times X_9$	77.84	1	77.84	0.19	0.6703
$X_7 \times X_{10}$	68.70	1	68.70	0.17	0.6891
$X_8 \times X_9$	17.19	1	17.19	0.04	0.8410
$X_8 \times X_{10}$	918.39	1	918.39	2.23	0.1575
$X_9 \times X_{10}$	333.07	1	333.07	0.81	0.3836
X_7^2	67507.68	1	67507.68	163.96	< 0.0001
X_8^2	16463.03	1	16463.03	39.99	< 0.0001
X_9^2	5811.67	1	5811.67	14.12	0.0021
X_{10}^2	4242.42	1	4242.42	10.30	0.0063
Residual	5764.18	14	411.73		
Cor Total	88811.18	28			

X_7 = glycerol content, X_8 = temperature, X_9 = drug weight, X_{10} = ultrasonication power, SS = sum of squares, DF = degrees of freedom, MS = mean square, R^2_A = adjusted R^2, and R^2_P = predicted R^2.

Table S5. Analysis of variance (ANOVA) for the Box-Behnken design model for RSA extraction.

	RSA				
R^2	$R^2 = 0.9069$, $R^2_P = 0.8137$, $R^2_A = 0.6187$				
Source	SS	DF	MS	*F* Value	*P*-value
Model	720.04	14	51.43	9.74	< 0.0001
Lack of Fit	44.41	10	4.44	0.60	0.7658
Pure Error	29.53	4	7.38		
X_7	296.15	1	296.15	56.07	< 0.0001
X_8	127.70	1	127.70	24.18	0.0002
X_9	4.04	1	4.04	0.76	0.3966
X_{10}	0.01	1	0.01	0.01	0.9904
$X_7 \times X_8$	3.97	1	3.97	0.75	0.4008
$X_7 \times X_9$	13.14	1	13.14	2.49	0.1371
$X_7 \times X_{10}$	0.89	1	0.89	0.17	0.6883
$X_8 \times X_9$	11.05	1	11.05	2.09	0.1700
$X_8 \times X_{10}$	8.44	1	8.44	1.60	0.2269
$X_9 \times X_{10}$	2.62	1	2.62	0.50	0.4927
X_7^2	180.02	1	180.02	34.08	< 0.0001
X_8^2	3.69	1	3.69	0.70	0.4173
X_9^2	25.50	1	25.50	4.83	0.0453
X_{10}^2	24.07	1	24.07	4.56	0.0509
Residual	73.94	14	5.28		
Cor Total	793.98	28			

X_7 = glycerol content, X_8 = temperature, X_9 = drug weight, X_{10} = ultrasonication power, SS = sum of squares, DF = degrees of freedom, MS = mean square, R^2_A = adjusted R^2, R^2_P = predicted R^2, and RSA = radical scavenging activity.

Table S6. Comparison of NADES and optimal extracts responses.

**Extract Name**	**TP (µg/mL)**	**Luteolin (µg/mL)**	**RSA (µL (extract/mL)**
1BGG-50-TP	658.19 ± 23.91[d,e,f,g]	174.26	1.39 ± 0.04[j,k,l]
2BGG-50-TP	702.48 ± 52.92[d,e,f]	48.65	4.18 ± 0.26[h]
GU-50-TP	776.45 ± 58.22[d]	201.08	6.04 ± 0.20[c,d,e,f]
PG-50-TP	1635.69 ± 60.15[c]	204.79	1.85 ± 0.02[j,k]
PG-50-TP-0.8[1]	1798.46 ± 37.25[b]	249.09	0.95 ± 0.03[l]
PG-50-TP-1.0[2]	2135.57 ± 64.32[a]	301.74	0.86 ± 0.04[l]
1BGG-25-TP	648.33 ± 29.09[e,f,g,h]	118.14	5.65 ± 0.28[e,f,g]
2BGG-25-TP	659.03 ± 49.60[d,e,f,g]	64.11	6.75 ± 0.48[c]
GU-25-TP	565.99 ± 47.29[g,h,i,j]	86.01	7.83 ± 0.22[b]
PG-25-TP	773.67 ± 54.40[d]	97.88	2.11 ± 0.01[j]
OPT-TP	740.00 ± 5.70[d,e]	152.55	3.20 ± 0.04[j]
1BGG-50-LUT	467.12 ± 2.88[j,k]	135.07	5.94 ± 0.26[c,d,e,f]
2BGG-50-LUT	536.95 ± 6.88[h,i,j]	96.36	6.79 ± 0.54[c]
GU-50-LUT	503.04 ± 46.71[j]	164.05	6.45 ± 0.14[c,d,e]
PG-50-LUT	721.76 ± 52.19[d,e]	176.50	0.74 ± 0.07[l]
1BGG-25-LUT	309.43 ± 16.90[l]	50.52	11.27 ± 0.74[a]
2BGG-25-LUT	356.80 ± 9.44[k,l]	74.90	5.78 ± 0.23[d,e,f,g]
GU-25-LUT	326.07 ± 17.92[l]	71.94	6.62 ± 0.21[c,d]
PG-25-LUT	584.85 ± 36.60[f,g,h,i,j]	84.60	1.19 ± 0.03[k,l]
OPT-LUT	360.83 ± 14.49[k,l]	156.23	8.23 ± 0.25[b]
1BGG-50-RSA	626.50 ± 36.91[e,f,g,h,i]	135.22	5.05 ± 0.22[g]

2BGG-50-RSA	626.39 ± 36.47[e,f,g,h,i]	36.05	6.17 ± 0.11[c,d,e]
GU-50-RSA	574.39 ± 1.76[g,h,i,j]	118.72	8.23 ± 0.51[b]
PG-50-RSA	775.27 ± 46.88[d]	93.41	1.03 ± 0.04[k,l]
1BGG-25-RSA	506.78 ± 36.52[j]	98.23	3.24 ± 0.15[i]
2BGG-25-RSA	502.94 ± 12.55[j]	33.69	3.35 ± 0.32[h,i]
GU-25-RSA	520.56 ± 31.91[i,j]	52.44	5.32 ± 0.11[f,g]
PG-25-RSA	542.67 ± 23.74[g,h,i ,j]	66.83	3.83 ± 0.01[h,i]
OPT-RSA	584.00 ± 28.12[g,h,i,j]	123.75	3.10 ± 0.09[i]

[1] extract prepared using 0.8 g of plant material,[2] extract prepared using = 1.0 g of plant material. TP = total phenolic content, RSA = radical scavenging activity. Values are average of three replications ± SD where applicable. [a-l] = differences between the extracts within a column (extracts not connected with the same capital letter are statistically different, Tukey post-test, $P < 0.05$). Abbreviations for optimized and NADES extracts are explained in Table 3 and subsection 3.3., respectively.
